# Using Dynamics of Eye Movements, Speech Articulation and Brain Activity to Predict and Track mTBI Screening Outcomes

**DOI:** 10.3389/fneur.2021.665338

**Published:** 2021-07-06

**Authors:** James R. Williamson, Doug Sturim, Trina Vian, Joseph Lacirignola, Trey E. Shenk, Sophia Yuditskaya, Hrishikesh M. Rao, Thomas M. Talavage, Kristin J. Heaton, Thomas F. Quatieri

**Affiliations:** ^1^Human Health and Performance Systems, MIT Lincoln Laboratory, Lexington, MA, United States; ^2^Counter-WMD Systems, MIT Lincoln Laboratory, Lexington, MA, United States; ^3^Advanced RF Techniques & Systems, MIT Lincoln Laboratory, Lexington, MA, United States; ^4^Electrical and Computer Engineering/Biomedical Engineering, Purdue University, West Lafayette, IN, United States; ^5^Military Performance Division, U.S. Army Research Institute of Environmental Medicine, Natick, MA, United States

**Keywords:** neurocognitive testing, eye tracking, speech, fMRI, fine motor coordination, resting state brain activity

## Abstract

Repeated subconcussive blows to the head during sports or other contact activities may have a cumulative and long lasting effect on cognitive functioning. Unobtrusive measurement and tracking of cognitive functioning is needed to enable preventative interventions for people at elevated risk of concussive injury. The focus of the present study is to investigate the potential for using passive measurements of fine motor movements (smooth pursuit eye tracking and read speech) and resting state brain activity (measured using fMRI) to complement existing diagnostic tools, such as the Immediate Post-concussion Assessment and Cognitive Testing (ImPACT), that are used for this purpose. Thirty-one high school American football and soccer athletes were tracked through the course of a sports season. Hypotheses were that (1) measures of complexity of fine motor coordination and of resting state brain activity are predictive of cognitive functioning measured by the ImPACT test, and (2) within-subject changes in these measures over the course of a sports season are predictive of changes in ImPACT scores. The first principal component of the six ImPACT composite scores was used as a latent factor that represents cognitive functioning. This latent factor was positively correlated with four of the ImPACT composites: verbal memory, visual memory, visual motor speed and reaction speed. Strong correlations, ranging between *r* = 0.26 and *r* = 0.49, were found between this latent factor and complexity features derived from each sensor modality. Based on a regression model, the complexity features were combined across sensor modalities and used to predict the latent factor on out-of-sample subjects. The predictions correlated with the true latent factor with *r* = 0.71. Within-subject changes over time were predicted with *r* = 0.34. These results indicate the potential to predict cognitive performance from passive monitoring of fine motor movements and brain activity, offering initial support for future application in detection of performance deficits associated with subconcussive events.

## 1. Introduction

Over the past decade, awareness and concern regarding the adverse effects of subconcussive head injuries has grown, particularly in the areas of sport and the military (1). With ongoing diagnostic challenges in concussion screening assessment, medical professionals face difficult real-time decisions about whether or not it is safe for athletes or Soldiers to continue performing after experiencing head impacts during play or on the battlefield. Mild concussions may not meet criteria for concussion with sufficient clarity to justify removing the individual from activity. In many cases overt symptoms of concussion may be fleeting or may only emerge over a period of several days following the insult (2, 3). At the same time, immediate removal from activity after concussion is associated with less time away from activity, a shorter symptomatic period, and better clinical outcomes (4). An athlete with an undiagnosed concussion who is not removed from the field may experience subsequent repetitive head impacts during play, with even mild mechanical head impacts exacerbating the original injury and complicating recovery (4). Over time, repeated subconcussive blows to the head during sports may also have cumulative and long-lasting neurological effects (5, 6).

Prior work in developing concussion screening technologies has primarily focused on active task-oriented protocols, such as cognitive tests and target-based eye tracking. For computerized assessment of neurocognitive function, ImPACT (Immediate Post-concussion Assessment and Cognitive Testing) is a widely used FDA-approved tool that provides baseline and post-injury assessment of visual memory, verbal memory, reaction time, and processing speed (7–9). Applied as part of a comprehensive clinical evaluation after a suspected neurotrauma, ImPACT has shown effectiveness in identifying concussions in high school and college athletes (10), as well as sub-concussive injuries during a sports season (11).

More recently, efforts have been made to identify measurement modalities that provide passive and unobtrusive measurements, thereby allowing longer-term, ambulatory monitoring. One of these modalities is eye tracking. In target-based eye tracking tests, individuals follow a moving target on a computer screen or visual display with their eyes while a screen-based eye tracker records gaze location as it changes within the space of the screen. Across numerous studies, eye movement dynamics have been identified as indicative of clinically-relevant neuromotor and cognitive deficits for a variety of neurological disorders, including concussions (12–14). Different eye tracking tasks are designed to capture different types of eye movements, including saccades and smooth pursuit, each governed by different neural circuits and thus providing distinct perspectives into an individual's neurological profile (15–17).

Despite the demonstrated benefits of ImPACT testing and target-based eye tracking tasks as assessment tools for concussion, both require active participation by the individual and are typically used only after a concussion is suspected via subjective impressions of decision makers on the field. In order to identify injuries that may occur during activity (or in real time), or to capture the cumulative effects of impacts over time, assessment technologies are needed that can passively monitor neurological state in a continuous, ongoing manner. A technology that provides such unobtrusive tracking of neurocognitive functioning in athletes could enable preventative interventions for athletes and others at elevated risk of concussive injuries.

Mobile eye tracking could provide passive, continuous neurological monitoring and evaluation. Unlike screen-based eye tracking tasks that follow a predetermined moving target on a computer screen, an assessment that uses mobile eye tracking would look for stand-alone, target-agnostic patterns in naturalistic eye movements that may be characteristic of concussion. Wearable eye tracking sensors such as Tobii (18) can potentially be integrated into athletic helmets and protective eye equipment for continuous monitoring. While there has been much prior research into target-based eye tracking assessments, so far little has been done to identify concussion indicators that can be measured in naturalistic eye movements (19).

An approach for measuring the complexity of coordinated movements during continuous monitoring has shown success in detecting the effects of a variety of neurological conditions on performance. In this *coordination complexity* approach, complexity is quantified by the dimensionality of a multivariate time series, as measured across multiple channels at multiple time delays. This is done by constructing a channel-delay correlation matrix from the time series data and using its rank-ordered eigenspectrum to quantify the dimensionality. A greater concentration of weight in the largest eigenvalues indicates lower complexity in the time series data because a larger fraction of the total variance can be explained using a smaller number of eigenvectors. A greater concentration of weight in the smaller eigenvalues, on the other hand, indicates greater complexity because a larger number of eigenvectors are required to explain a given fraction of the total variance (20).

This approach has been used previously to differentiate mild traumatic brain injury (mTBI) subjects from control subjects based on gait movements (21), autistic subjects from control subjects based on hand drawing movements (22), and Parkinson's disease (PD) subjects from control subjects based on small-magnitude wrist movements (23). Whether neurological degradation results in greater or lower complexity depends on the specifics of the underlying behavior. Subjects with mTBI produced gait torso movements with greater complexity than normal subjects. Subjects with autism, on the other hand, produced stylus movements during a drawing task with reduced complexity. Subjects with PD produced small-magnitude wrist movements with lower complexity than control subjects during free-living conditions.

Explanations for the differential effects of neurological degradation on these different behavioral tasks are provisional, but center on the consequences of reduced feedforward and feedback neuromotor control. Lower feedback control produces ataxic gait, which is characterized by wobbliness and loss of balance (24, 25), and hence an increase in complexity of torso accelerations. Reduced feedforward and feedback control in autism result in fine movement accelerations with lower complexity, but at the cost of reduced precision in handwriting (26, 27). Loss of feedback control in PD subjects reduces stabilizing movements, resulting in postural accelerations with lower complexity and dimensionality (28, 29). The sensitivity of coordination complexity features to these changes motivates the use of this feature approach to analyze the associations between continuous smooth pursuit eye movements and ImPACT performance.

Analysis of speech is also promising as a passive method for detecting neurological trauma. Features extracted from vowels recorded on a mobile device by athletes participating in a boxing match were found to predict the presence of a concussion with an accuracy of 98% (30). Another study was able to achieve an area under the receiver operating characteristic curve (AUC) of 0.86 in detecting mTBI by using speech features obtained from recordings of sentences and words collected from high school athletes (31). Speech production requires the highly complex coordinated movement of speech articulators. The coordination complexity feature approach has been widely used in analysis of continuous speech. In particular, measures of reduced complexity in articulatory coordination, derived from speech signals, have been used to detect mTBI (21), to detect cognitive performance changes in high school athletes during a sports season (32), and to detect reduced cognitive processing speed in mTBI patients (33). These studies highlight the potential for speech tasks as a non-invasive screening tool for mTBI and return-to-activity readiness.

Measures of functional connectivity in the brain have shown effectiveness in estimating the efficiency of cognitive processing, not only during active performance of specific cognitive tasks (34, 35), but also in examining resting state brain activity (5). Methods for assessing connectivity typically rely on global graph-based measures of functional connectivity, as in Ahmadlou et al. (35), or on local measures of correlated signals between specific brain regions, as in Johnson et al. (5). The coordination complexity feature approach is a global measure of functional connectivity, based on a quantification of the structure of correlations across all measured brain regions at multiple time lags. As described in section 3.4, this approach essentially quantifies the complexity of vector autoregressive models of fMRI time series data. Vector autoregressive modeling has been widely used in fMRI analysis (36), and variants of the coordination complexity approach have been applied to EEG data for analyzing and predicting epileptic seizures (37–40) and for assessing cognitive load and performance (41, 42). A variant of coordination complexity features has been compared directly to standard graph complexity measures (average path length and average degree) and generated higher accuracy in predicting cognitive performance on a working memory task (42). While robust wearable sensor technologies for monitoring functional brain activity have begun to emerge (43), these technologies are less mature than eye tracking or speech in providing a robust wearable sensing capability. However, establishing their potential use as predictive markers could motivate further investments in direct sensing of functional connectivity for detecting neurological state change.

In this paper, we explore the efficacy of using multimodal measures of fine motor coordination and resting state brain activity to predict cognitive performance outcomes, as quantified by six ImPACT composite scores, across a season of play in high school athletes. The relationship of these measures to ImPACT performance is assessed, both using global correlations between features derived from these measures and ImPACT performance, and using a trained model to predict ImPACT performance out of sample. While this study utilizes laboratory data collected during naturalistic or quasi-naturalistic tasks, our intent is to establish a preliminary prediction model, using measures that are amenable to passive field data collection, that will serve as the foundation for future implementation in ambulatory, free living conditions.

## 2. Data Set

### 2.1. Subject Enrollment

Twenty-five high school male football players and seven high school female soccer players, ages 15–18, were enrolled in the study, which was conducted by Purdue University. All participants provided written informed consent and procedures were approved by both the Purdue Institutional Review Board and the MIT Committee on Use of Humans as Experimental Subjects. There was a total of 32 subjects and 114 assessment sessions. Speech data was obtained from all of these sessions. Usable eye tracking data was obtained from 31 subjects and 93 sessions. The reason some eye tracking data was unusable is described in section 3.1. fMRI data was measured from 30 subjects and 87 sessions. Assessments were analyzed only if all three modalities were obtained in the same session. These resulted in the analysis of 28 subjects and 68 sessions of data, including 22 football players (52 sessions) and 6 soccer players (16 sessions). None of these subjects had been diagnosed with concussions at the time of the assessments.

Pre-season and in-season activity involved the subjects' respective sport practices, drills, and games. Additionally, some subjects participated in other sports during the post-season assessments. ImPACT scores, as well as the eye tracking, speech, and fMRI sensor measurements were all recorded on the same day. Sessions occurred at five assessment points in the late Summer and Fall 2012 sports season: Pre-Season, Early-Season, Late-Season, and two Post-Season assessment points. Table 1 shows, for each of these assessments, the number of subjects that were analyzed, the mean assessment date across subjects, and the standard deviation (in days) of assessment dates. The average within-subject interval between consecutive sessions was 33.6 days.

**Table 1:** Dates of analyzed assessments in 2012 sports season.

**Assessment**	**# Subjects**	**Mean date**	**Std. Date**
		**(mm/dd)**	**(days)**
Pre	21	07/27	5.5
Early	20	08/27	11.5
Late	14	09/25	8.5
Post 1	8	10/28	6.7
Post 2	5	11/28	10.3

### 2.2. ImPACT Scores

In each recording session, subjects completed the computerized ImPACT test. For the purposes of this study, ImPACT scores were used as an indicator of cognitive health and did not influence return to play decisions. Subjects were administered the online ImPACT test, version 2.1, to monitor changes in neurocognitive functioning during the season. The ImPACT test yields six component measures: verbal memory, visual memory, visual motor, reaction time, impulse control, and total symptom composites. A composite score is calculated from various relevant metrics within each component (44). The verbal memory composite includes word, symbol, and letter recall tests and provides an evaluation of attention, learning, and verbal memory. The visual memory composite consists of object recall tests to target visual attention, scanning, learning, and memory skills. The visual motor composite assesses visual processing, learning, memory, and motor response speed with metrics from the object and letter recall tests. The reaction time composite incorporates parameters from the object recall test as well as color and symbol matching tests to evaluate response speed. The impulse control composite evaluates the number of errors committed during the object recall and color match tests. This metric is used in the interpretation of other scores and overall test validity. The total symptom composite is a sum of values from a scaled set of concussion-related symptoms reported by the subject.

## 3. Measurements

The goal of feature extraction was to characterize the complexity of fine motor control and of brain activity from sensor measurements. It is hypothesized that neurological deficits associated with concussion can cause changes in such complexity. Complexity is quantified from correlation patterns among multiple channels of time series measures in a sensing modality, such as from eye tracking and speech data (20). These same complexity features can also be extracted from time series measurements of brain activity.

### 3.1. Eye Tracking Signals

Eye movements were recorded during a smooth pursuit task, in which subjects were instructed to follow a target that moved in a circular pattern around the screen. The target appeared on the screen for roughly 1.5 s followed by a 0.5 s break before reappearing. The target made 35 cycles in just over 2 min. The position of the subject's gaze (i.e., x and y position on the screen) was tracked using a SR Research Eyelink 1,000 eye tracker, running at 1,000 Hz.

The *x, y* eye tracking coordinates were analyzed within between-blink time segments. Blinks were quantified as when the sensor did not report numerical values, or when the absolute value of the sum of discrete-time derivatives across the *x* and *y* coordinates was greater than a threshold of 100. Eye tracking segment boundaries were separated from the nearest detected blinks by a gap of 50 ms. The resulting between-blink segments were required to have a duration of at least 10 s to be retained for further analysis. In 21 of the sessions, there were no between-blink segments of at least 10 s duration found, and so eye tracking data was not analyzed in these sessions. This segmentation was followed by smoothing, which was done independently in the *x*- and *y*-coordinate time series using a time-domain Gaussian filter with a standard deviation of 21 ms.

### 3.2. Audio Signals

The subject's vocal data was captured with an Audio-Technica ATM73A Fixed Charge Condenser head worn microphone. Vocal data included a read speech task called the Grandfather passage, which was developed to elicit a standardized phonetically balanced speech sample (45). Speech utterances had a mean duration of 51.5 s with standard deviation of 9.2 s. Each audio recording was transformed into three formant frequency trajectories, which represent the resonant frequencies of the vocal tract. The formant frequencies change over time as speech articulators (lips, tongue, etc.) move. The three lowest formant frequencies were tracked and extracted every 10 ms from the audio signal using the KARMA software tool (46). Delta-formants (dFormants), the discrete-time derivatives of the formants, were also computed.

### 3.3. fMRI Signals

Functional magnetic resonance imaging (fMRI) measures brain activity by detecting changes associated with blood flow. When an area of the brain is active, blood flow to that region typically increases. Signals were derived from resting state fMRI scans of the subjects. The MRI scanner used was a General Electric 3T Signa HDx Scanner and the scans were acquired using a gradient-echo planar sequence. Resting state brain activity was recorded for an average of 580 s per subject (6).

The signals are the time-series of the blood oxygenation level dependent (BOLD) responses for the regions of interest (ROI). This provides a measure of functional activity of the brain for the duration of the scan. Preprocessing of the resting state fMRI and accompanying structural MRI data (for Atlas registration) was performed with the toolkit CONN (47). This step involved bandpass filtering (0.01 to 0.10 Hz). The Harvard-Oxford cortical (cort-maxprob-thr25–2 mm) and subcortical (sub-maxprob-thr25–2 mm) structural atlases (https://neurovault.org/images/1699/) were used to aggregate fMRI measurements into a standard set of time series, resulting in 48 cortical ROI time series and 21 subcortical ROI time series, generated at 0.5 Hz. Tables 2, 3 list the subcortical and cortical ROIs.

**Table 2:** Subcortical fMRI regions of interest (ROIs).

**#**	**ROI**	**#**	**ROI**
1	left cerebral white matter	12	right cerebral white matter
2	left cerebral cortex	13	right cerebral cortex
3	left lateral ventrical	14	right lateral ventrical
4	left thalamus	15	right thalamus
5	left caudate	16	right caudate
6	left putamen	17	right putamen
7	left pallidum	18	right pallidum
8	brain-stem	19	right hippocampus
9	left hippocampus	20	right amygdala
10	left amygdala	21	right accumbens
11	left accumbens		

**Table 3:** Cortical fMRI regions of interest (ROIs).

**#**	**ROI**	**#**	**ROI**
1	frontal pole	25	frontal medial cortex
2	insular cortex	26	juxtapositional lobule cortex
3	superior frontal gyrus	27	subcallosal cortex
4	middle frontal gyrus	28	paracingulate gyrus
5	inferior frontal gyrus, pars triangularis	29	cingulate gyrus, anterior division
6	inferior frontal gyrus, pars opercularis	30	cingulate gyrus, posterior division
7	precentral gyrus	31	precuneous cortex
8	temporal pole	32	cuneal cortex
9	superior temporal gyrus, anterior division	33	frontal orbital cortex
10	superior temporal gyrus, posterior division	34	parahippocampal gyrus, anterior division
11	middle temporal gyrus, anterior division	35	parahippocampal gyrus, posterior division
12	middle temporal gyrus, posterior division	36	lingual gyrus
13	middle temporal gyrus, temporooccipital part	37	temporal fusiform cortex, anterior division
14	inferior temporal gyrus, anterior division	38	temporal fusiform cortex, posterior division
15	inferior temporal gyrus, posterior division	39	temporal occipital fusiform cortex
16	inferior temporal gyrus, temporooccipital part	40	occipital fusiform gyrus
17	postcentral gyrus	41	frontal operculum cortex
18	superior parietal lobule	42	central opercular cortex
19	supramarginal gyrus, anterior division	43	parietal operculum cortex
20	supramarginal gyrus, posterior division	44	planum polare
21	angular gyrus	45	heschls gyrus
22	lateral occipital cortex, superior division	46	planum temporale
23	lateral occipital cortex, inferior division	47	supracalcarine cortex
24	intracalcarine cortex	48	occipital pole

Summary statistic features computed from the cortical and subcortical ROIs were the within-channel variances of the signals over the duration of the scan. These features quantify the average spatial distribution of subcortical and cortical activity. In addition, coordination complexity features (described below), which summarize the spatiotemporal correlation structure of the ROI time series, were computed independently from the cortical and subcortical ROIs.

### 3.4. Coordination Complexity Features

Coordination complexity features represent the correlation structure of multivariate signals across a set of time delays. These features are the eigenspectra of *channel-delay* correlation matrices. Here, *channel* refers to the signal time series from each modality: smoothed *x, y* eye tracking coordinates, speech formant or delta-formant trajectories, and subcortical or cortical fMRI ROI time series. *Delay* refers to time delays at which correlations are computed both between and within the channels. Channel-delay correlation matrices are simply correlation matrices of an expanded number of time series that are obtained via time-delay embedding, which is the creation of new time series channels that are time-shifted versions of the original signals. The eigenspectra of the channel-delay matrices represent the complexity of a vector autoregression model for predicting future values of a (z-scored) multivariate time series, given the finite history determined by the number of time delays (embedding dimensionality) (36). Thus, they capture the intrisic dimensionality of multivariate time series signals over the range of temporal frequencies sampled by the time delays.

Specifically, a channel-delay correlation matrix is computed as

(1) $$\begin{array}{r} \text{R}=\left[\begin{array}{ccc} R_{1,1} & \ldots & R_{1,M}\\ \vdots & \ddots & \vdots \\ R_{M,1} & \ldots & R_{M,M} \end{array}\right] \end{array}$$

where *M* is the number of low-level feature channels. Each submatrix *R*_*c*_1_, *c*_2__ contains the set of correlations between channels *c*_1_ and *c*_2_,

(2) $$\begin{array}{r} R_{c_{1},c_{2}}={\left[\begin{array}{ccc} r_{1,1} & \ldots & r_{1,N}\\ \vdots & \ddots & \vdots \\ r_{N,1} & \ldots & r_{N,N} \end{array}\right]}_{c_{1},c_{2}} \end{array}$$

where *N* is the number of delays per channel and [*r*_*d*_1_, *d*_2__]_*c*_1_, *c*_2__ is the correlation between channel *c*_1_ at delay *d*_1_ with channel *c*_2_ at delay *d*_2_. For all signal modalities, the number of delays is *N* = 15. The spacing between successive delays is 0.5 s for eye tracking, 0.03 s for formants and delta-formants, and 2 s for cortical and subcortical fMRI.

**Figure 1:**
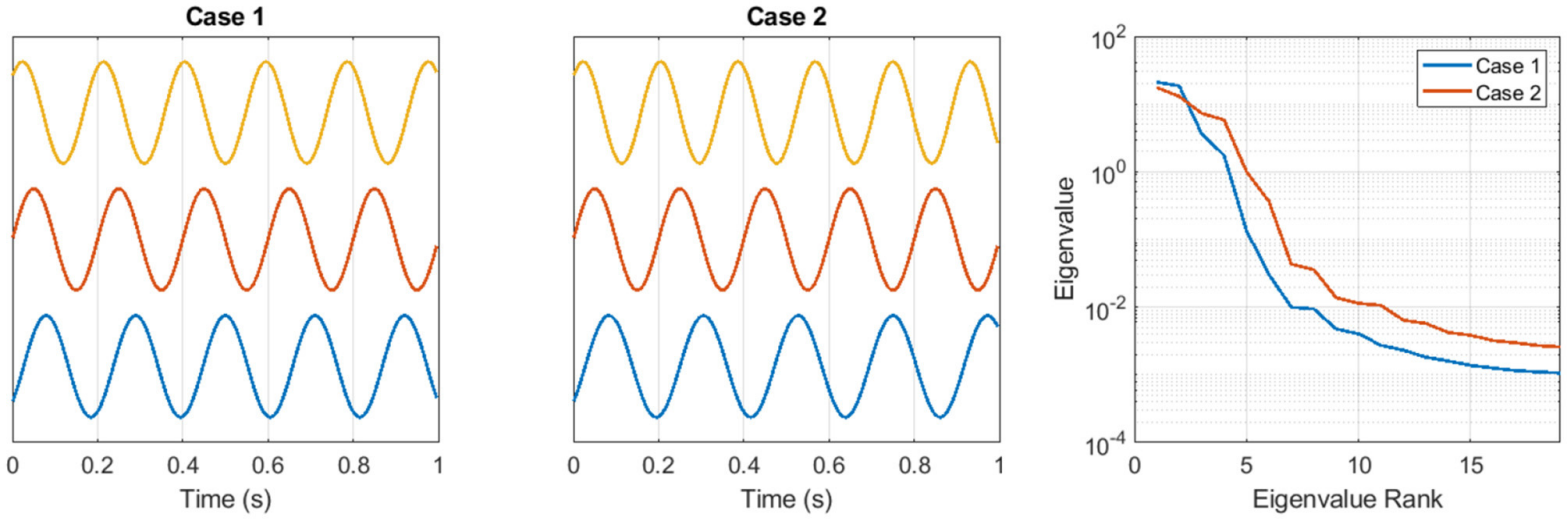
Two sets of artificial time series are used to illustrate the coordination complexity feature approach. See text for details.

Figure 1 shows an example of the coordination complexity features. Two artificial time series are generated from the equation *y*_*i*_ = sin(2π*f*_*i*_*t* + ϕ_*i*_), for channels *i* = 1, 2, 3, across the time interval 0 < *t* < = 1. The frequencies in Case 1 are *f*_1_ = 4.75, *f*_2_ = 5.0, *f*_3_ = 5.25 and in Case 2 are *f*_1_ = 4.5, *f*_2_ = 5.0, *f*_3_ = 5.5. The phase offsets in Case 1 and Case 2 are identical. The signals in Figure 1 are shown vertically offset for clarity. Because Case 2 contains a broader range of frequencies, the phase relationships across the three channels are more variable. In that sense, the set of multivariate time series in Case 2 is more complex. This greater complexity is quantified in Figure 1right by the eigenspectra of channel-delay matrices that are constructed using *N* = 15 delays with a delay spacing of 0.005*s*. The small eigenvalues from Case 2 are larger than the small eigenvalues from Case 1.

For speech and fMRI features, a single matrix and eigenspectrum was computed from the entire time series collected in a recording session. For eye tracking features, the time series was segmented into multiple between-blink segments. A correlation matrix was computed from each 10-s frame within the segments, with 5 s of overlap between successive frames. The eye tracking eigenvalues in each rank were averaged across the multiple frames in each recording session.

### 3.5. ImPACT Latent Factor

Table 4 shows the matrix of Spearman correlations between the six different ImPACT composite scores, computed across all subjects and time. Note that higher scores on ImPACT reflect better cognitive performance outcomes. The first four composites are highly correlated with each other while being uncorrelated with the final two composites (impulse control and subjective symptoms). The first four ImPACT composites are verbal memory, visual memory, visual speed, and reaction speed. These composites measure aspects of cognitive, perceptual and motor processing that are known to correlate with cognitive ability (48). *Reaction speed* is the reciprocal of the ImPACT reaction time composite score. Reaction speed is used in this paper because it has the same sign of correlation as the verbal memory, visual memory, and visual speed composites. The six ImPACT composites were z-scored and then the first principal component was computed. This principal component has a weighting vector of (0.49, 0.51, 0.49, 0.51, 0.01, −0.07), and thus is essentially an average of the normalized scores of the first four ImPACT composites. Thus, these four composites have essentially equal contribution to a latent factor related to cognitive ability that explains the largest component of variation in ImPACT scores. This latent factor has a mean of zero and standard deviation of 1.62. There were no group level trends in the latent factor over time. No statistically significant differences were found at the group level between the latent factor values in any of the five assessment points (Pre, Early, Late, Post 1, Post 2) listed in Table 1 (*p*>0.05).

**Table 4:** Table of Spearman correlations between all six ImPACT composite scores.

**ImPACT Composites**	**1**.	**2**.	**3**.	**4**.	**5**.	**6**.
1. Verbal memory		0.65	0.49	0.47	–0.08	–0.17
2. Visual memory	0.65		0.54	0.58	–0.07	–0.04
3. Visual speed	0.49	0.54		0.69	–0.02	–0.05
4. Reaction speed	0.47	0.58	0.69		0.10	0.01
5. Impulse control	–0.08	–0.07	–0.02	0.10		–0.03
6. Subjective symptoms	–0.17	–0.04	–0.05	0.01	–0.03	

## 4. Prediction Model

**Figure 2:**
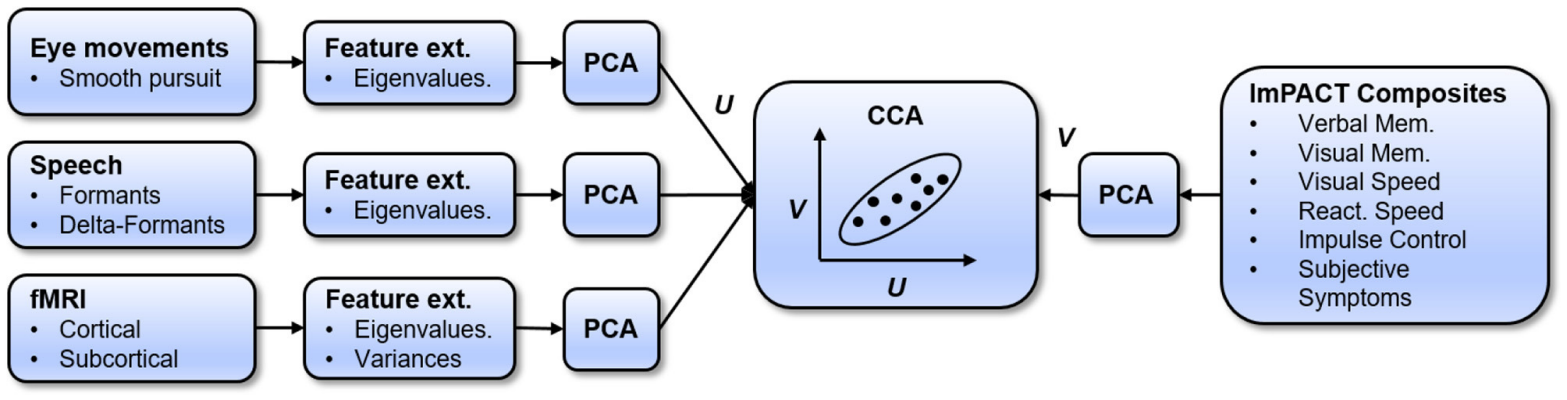
Diagram of processing pipeline.

The prediction model, which learns a mapping between sensor measures and ImPACT composite scores, is diagrammed in Figure 2. The model uses a two-level mapping. The first step is to use principal component analysis (PCA) to reduce the dimensionality of the high-level feature sets and of the ImPACT composites, in a way that maximally preserves their variance for a given dimensionality. Dimensionality reduction is necessary to avoid learning an overfit model that generalizes poorly. The next step is to use canonical correlation analysis (CCA) (49) to map the feature-based principal components (PCs) to the ImPACT-based PCs. CCA is a flexible regression approach because it allows multivarate PCs on both sides of the mapping, finding the linear mapping that maximizes total input/output correlation.

These two steps are used within a leave-one-subject-out (LOSO) cross-validation framework, which determines if the learned models can generalize to new subjects. An additional level of nested cross-validation within each training fold is also used to select the best performing number of PCs to extract from each sensor-based feature vector, because the most effective PC dimensionality varies with feature type. The prediction model and training methodology are described in detail below.

### 4.1. Principal Component Analysis

PCA is used to project feature vectors to lower dimensional spaces, with the first dimension capturing the largest covariance direction, and subsequent dimensions capturing the largest orthogonal residual covariances. The feature vectors that are input to PCA are first z-scored so that all feature dimensions have unit variance, and are weighted equally.

Let *X* denote the set of z-scored features (from eye tracking, speech, or fMRI) and *Y* denote the z-scored ImPACT scores. Then, PCA is used to generate lower-dimensional feature vectors $\hat{X}\left(N\times m_{X}\right)$ and outcomes Ŷ (*N* × *m*_*Y*_), where *N* is the number of observations (i.e., sessions), *m*_*X*_ is the feature PC dimensionality, and *m*_*Y*_ is the ImPACT PC dimensionality.

For each feature modality, the number of PC features is selected (using nested leave-one-subject-out (LOSO) cross-validation) that produces the best canonical correlation in the latent space on held-out data within each training fold. The cross-validation procedure is described in section 4.3. PC feature vectors generated from multiple feature sets are fused via vector concatenation.

Ŷ is the matrix of PCA values representing ImPACT composite scores. Based on the analysis of correlation structure of ImPACT composites, described in section 3.6, we set *m*_*Y*_ = 1 for the main analysis in this paper, to explore correlations with the latent cognitive factor. Then, an examination of the effect of varying *m*_*Y*_ is also conducted.

### 4.2. Canonical Correlation Analysis

Next, CCA is used to find projection matrices *A* (*m*_*X*_ × *m*_*X*_) and *B* (*m*_*Y*_ × *m*_*Y*_) that map $\hat{X}$ and Ŷ into a latent space that maximizes their correlation,

(3) $$\begin{array}{r} U=\left[\hat{X}-E\left(\hat{X}\right)\right]\cdot A \end{array}$$

(4) $$\begin{array}{r} V=\left[\hat{Y}-E\left(\hat{Y}\right)\right]\cdot B \end{array}$$

with the first dimension capturing the largest correlation, and subsequent dimensions capturing the largest residual correlations.

After this model is trained, it can be used to generate an estimate of the outcome feature vector *y* (ImPACT composite scores) based on an associated input feature vector *x* (coordination features and/or average fMRI features). This is done by inverting the above PCA and CCA mappings between the outcome feature space and the latent space.

### 4.3. Cross Validation

Nested LOSO cross-validation is conducted to provide an unbiased estimate of prediction accuracy on out-of-sample data. Predictions on all sessions for each subject are made based on a model trained on the remaining subjects. Within that training set, an additional level of LOSO cross-validation is done to select the best *x*_*M*_, based on the Spearman correlation of *U* and *V* generated on the union of test folds within that training set. In this selection process, *x*_*M*_ values in the range 1–7 are considered. The maximum number of allowed PCs is set to the number (7) that explains at least 90% of the variance in each of the coordination features.

### 4.4. Fusing Sensor Modalities

Accuracy in predicting cognitive performance can potentially be improved by fusing the information from multiple feature modalities. In our approach (see Figure 2), the sensor modalities are fused by concatenating their PCA feature vectors. The PCA dimensionality is selected within each feature set independently, using nested cross-validation. Then, sensor modalities are combined by concatenating their PCA feature vectors, using the PCA dimensionality in each cross-validation fold that was selected based on nested cross-validation. For each set of fused feature PCA vectors, a new CCA model is learned. The accuracy of this model is measured in predicting, on held-out test subjects, the ImPACT latent cognitive factor and the ImPACT composites.

### 4.5. Varying ImPACT Dimensionality

It is possible that more accurate predictions of the ImPACT composites can be obtained by expanding the dimensionality in the CCA latent space beyond the single latent factor represented by the first ImPACT principal component. This is explored by holding the number of feature PCs constant, varying the number of ImPACT PCs, and assessing the accuracy in predicting the ImPACT composite scores on held out subjects (via cross-validation). In section 4.3, the number of feature PCs was selected in each cross-validation fold while holding fixed *m*_*Y*_ = 1. An additional analysis is now done in which the feature PC dimensionality is held constant and *m*_*Y*_ is varied. The effect on accuracy in predicting ImPACT composite scores on held-out subjects is then measured.

### 4.6. Predicting Within-Subject Changes

Some of the ability to predict differences in ImPACT outcomes based on eye tracking, speech, and brain activity is due to inter-subject feature variance that correlates with outcome variance. But, an important use case for this technology is the tracking of intra-subject changes over time.

This capability is evaluated by determining if changes in within-subject predictions follow the same pattern as between-subject predictions. This is done by using a single global prediction model that is trained using the most commonly selected number of feature PCs per fold, based on the cross-validation procedure. Then, the correlations of changes (over successive sessions) in the feature-based latent factor *U* were computed against changes in the ImPACT-based latent factor *V*. In other words, correlations were computed between *dU* and *dV*, where *dU* = *U*_*i*+1_ − *U*_*i*_, and *dV* = *V*_*i*+1_ − *V*_*i*_, and where *i* indexes the session number for each subject. This analysis included 21 subjects with a total of 40 *dU* and *dV* data points from successive sessions.

A possible limitation of this approach is range restriction. It is possible that *dU* and *dV* are as strongly correlated over their full possible range of variation as *U* and *V*, but due to a limited range of variation in the current data set (range restriction), *dU* and *dV* will appear to have a smaller correlation. A standard adjustment to the correlations for range restriction was thus also computed (50, 51).

## 5. Results

### 5.1. Correlations Between Features and ImPACT

**Figure 3:**
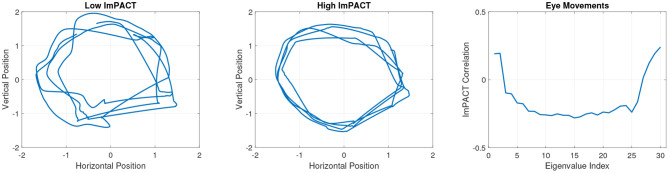
Session 1 eye tracking trajectories from a subject with a low latent ImPACT score **(left)** and a subject with a high latent ImPACT score **(middle)**. Correlations of eye tracking eigenvalues with latent ImPACT score from all data **(right)**. Note that a larger ImPACT latent score reflects better performance outcomes on the first four ImPACT composites (see section 3.5).

An exploratory analysis was conducted of the pattern of correlations between coordination complexity features and the ImPACT latent cognitive factor. Strong correlations (|*r*| > 0.3) were found with features from all three sensor modalities. Figure 3 shows an example of segmented eye tracking data from the first session of two subjects, one with a low score of –1.53 in the latent cognitive factor (subject 1, left) and one with a high score of 2.62 (subject 2, middle). The eye tracking traces of subject 1 show lower precision and consistency than those of subject 2. Coordination complexity features were extracted from the eye tracking segments by constructing channel-delay correlation matrices, as described in Equations (1, 2), and extracting the eigenspectra of the matrices.

The greater precision in smooth pursuit movements among subjects with higher cognitive ImPACT scores, as exemplified in the two tracings in Figure 3, is quantified in the eigenspectra. Figure 3right shows Spearman correlations of the eigenspectra (sorted in descending order of magnitude) with the latent cognitive factor. Spearman correlations were used because they are more robust to outliers than Pearson correlations. Before computing the correlations, the average eigenvalue per session at each rank was computed across the multiple 10 s segments.

**Figure 4:**
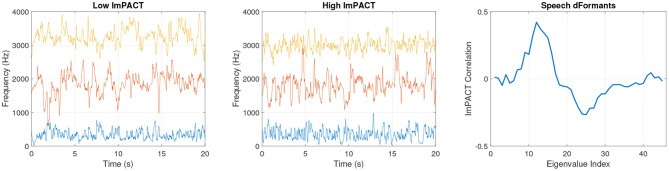
Session 1 formant frequencies from a subject with a low latent ImPACT score **(left)** and a subject with a high latent ImPACT score **(middle)**. Correlations of delta-formant eigenvalues with ImPACT latent score from all data **(right)**.

Figure 4 shows speech formant tracks that were obtained from subjects 1 and 2 in their first session. The formant tracks are illustrated from the first 20 s of the Grandfather passage. The formants produced by subject 2 have greater high-frequency fluctuations relative to low-frequency fluctuations over time. This reflects greater complexity of movement coordination in motor articulation. The association of higher articulatory complexity in subjects with higher cognitive scores is reflected in the pattern of Spearman correlations of the delta-formant eigenvalues with the ImPACT latent cognitive factor in Figure 4right. These correlations were computed across all subjects and sessions.

**Figure 5:**
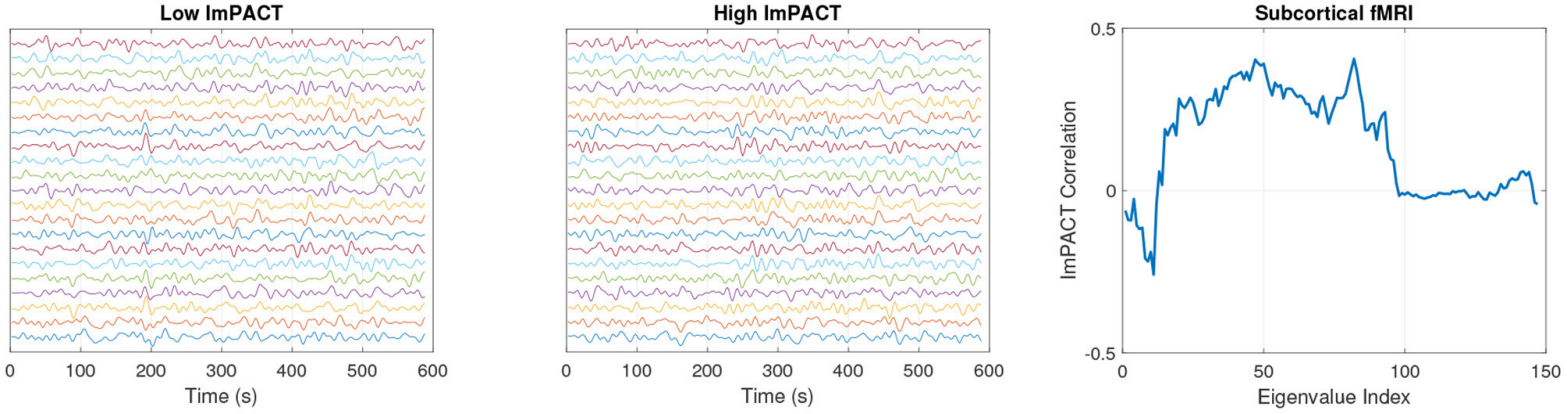
Session 1 normalized (z-scored) subcortical fMRI time series from a subject with a low latent ImPACT score **(left)** and a subject with a high latent ImPACT score **(middle)**. Correlations of subcortical fMRI eigenvalues with latent ImPACT score from all data **(right)**.

Figure 5 shows subcortical fMRI time series from subjects 1 and 2 during their first session. The time series have been z-scored and vertically offset in ascending order (see Table 2) so that their temporal dynamics can be seen more easily. The fMRI time series for subject 2 show a greater level and variety of correlations in high amplitude fluctuations between different channels. These patterns are difficult to interpret to the naked eye. However, as shown in Figure 5right, positive correlations with the latent cognitive factor across a range of eigenvalues indicate an increase in fMRI complexity with cognitive performance.

### 5.2. Predictions From Individual Feature Sets

First, we assess how well each feature set predicts the latent cognitive factor. This is done by computing the correlations, on held-out test subjects, of sensor-based *U*-values with ImPACT-based *V*-values. Table 5 summarizes these results, showing Spearman correlations between *U* and *V* with each of the feature sets as input. The number of PCA features that was most commonly selected from the training set folds is also shown. All feature sets produce positive correlations of *U* and *V* except fMRI cortical variance values.

**Table 5:** Latent space correlations between *U* and *V* using individual feature sets as input (*n* = 68).

**Modality**	**Feature**	**PCA**	****U**, **V****	
		**#**	***r***	***p***
1. Eye	Tracking eig.	7	0.45	0.000
2. Speech	Formant eig.	4	0.21	0.088
	dFormant eig.	2	0.35	0.003
3. fMRI	SubC eig.	4	0.39	0.001
	Cort eig.	7	0.28	0.020
	SubC variance	2	0.30	0.014
	Cort variance	1	–0.04	0.761

*Correlations are obtained from the union of LOSO cross-validation test folds*.

### 5.3. Predictions From Fused Feature Sets

Next, the effect of fusing feature sets via concatenation of their PCA vectors is explored. First, rows 1–3 of Table 6 show Spearman correlations obtained from each feature modality. The Speech results include fusion between formant and delta-formant features, and the fMRI results include fusion between subcortical and cortical features, and between coordination complexity (eigenvalue) features and variance features. The accuracy is also shown of predictions of the four ImPACT composites, Verbal Memory, Visual Memory, Visual Motor Speed, and Reaction Speed, which are the contributors to the latent cognitive factor. Correlations with the remaining two ImPACT composites (Impulse Control and Subjective Symptoms) are small in all cases, and not shown. Eye tracking and fMRI produce stronger correlations than Speech. Correlations with the latent factor are generally stronger than those with the individual composite scores.

**Table 6:** Latent space correlations between *U* and *V* given individual feature modalities and fused modalities as input (*n* = 68).

**Modality**	**U, V**	**Verbal**	**Visual**	**Visual**	**React**.
		**Mem**.	**Mem**.	**Speed**	**Speed**
	**r**	**r**	**r**	**r**	**r**
1. Eye	0.45	0.35	0.30	0.13	0.30
2. Speech	0.26	0.28	0.18	0.22	0.24
3. fMRI	0.49	0.34	0.42	0.31	0.33
1, 2	0.51	0.42	0.43	0.23	0.38
1, 3	0.60	0.47	0.54	0.35	0.50
2, 3	0.58	0.38	0.48	0.53	0.49
1, 2, 3	0.71	0.55	0.64	0.51	0.57

*Also, correlations of predictions with true scores for the four cognitive ImPACT composites. Correlations are obtained from the union of the LOSO cross-validation test folds*.

**Figure 6:**
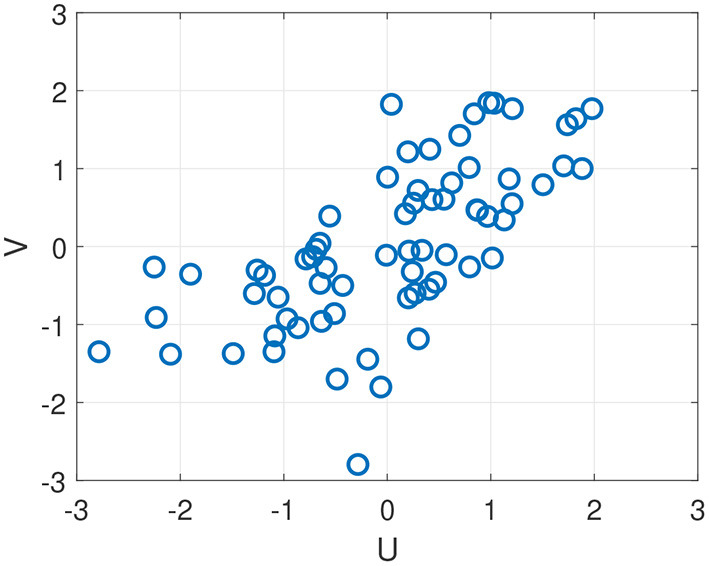
Scatter plot of *U* and *V* values obtained on out-of-sample test data using all three sensor modalities, resulting in Spearman correlation of *r* = 0.71 (see Table 6, bottom row).

Rows 4–7 show correlations obtained by fusing across different combinations of feature modalities. The strongest combination is obtained by fusing all three modalities (row 7), resulting in a correlation in the latent space of *r* = 0.71 and correlations with the four composites ranging between *r* = 0.51 and *r* = 0.64. The correlations with the remaining two composites (Impulse Control and Subjective Symptoms) are small and not shown. Figure 6 shows a scatter plot of *U* and *V* values on all test subjects and sessions for the strongest fused system (*r* = 0.71). Notice that, on the lower left quadrant of the scatter plot, the correlation between predicted scores *U* and true scores *V* is weak. This indicates that the model loses it's ability to predict differences in performance below a certain performance level.

### 5.4. Varying the Number of ImPACT Principal Components

**Figure 7:**
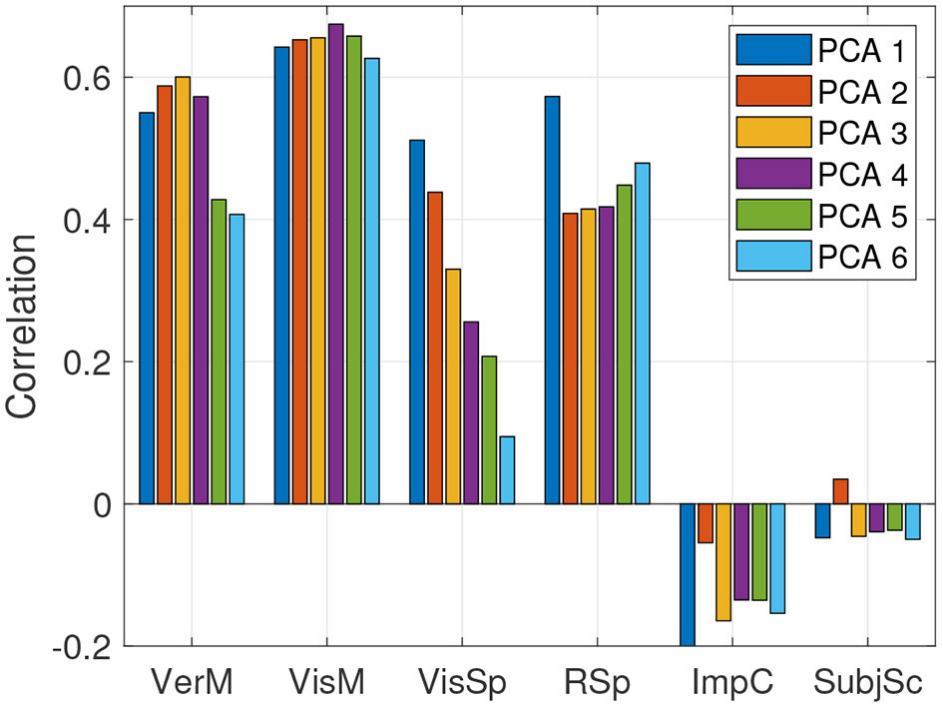
Correlations of predicted vs. true ImPACT composite scores on held-out test data, based on fusing all three sensor modalities, using different numbers of PCA components for mapping the ImPACT composites into the CCA latent space.

The predictions in sections 5.2, 5.3 were done using a single ImPACT principal component and hence a CCA latent space dimensionality of one. It was shown how well sensor measurements predict outcomes in the latent space along a single cognitive dimension, and how well these latent space predictions map back into the ImPACT composite scores. We explored if utilizing additional latent space dimensions could provide additional value, quantified by accuracy in predicting the ImPACT composites. We therefore varied the number of ImPACT principal components (and hence the number of CCA latent space dimensions) while fixing the number of feature-based principal components to the most common number per feature set, as shown in Table 5. The effect on accuracy in predicting the ImPACT composites is shown in Figure 7. Increasing the ImPACT PCA dimensionality slightly increases accuracy of predicting the Verbal Memory and Visual Memory composites, but at the cost of lower accuracy in Reaction Speed and particularly Visual Speed. Predictions of Impulse Control and Subjective Symptoms are small and statistically insignificant (*p*>0.05) in all cases, indicating that the three sensor modalities are not useful indicators of these components of the ImPACT test.

### 5.5. Predicting Within-Subject Changes

Within-subject changes in cognitive performance over time can be predicted based on changes in sensor features. Table 7 shows the accuracy of these predictions via Spearman correlations of within-subject changes in the latent space, between *dU* and *dV*, in successive sessions (21 subjects, 40 session pairs). To obtain this result, a single prediction model was trained using all training data (28 subjects, 68 sessions). This was done using the PCA dimensionality per feature set shown in Table 5. The within-subject correlations are smaller than the between subject correlations, but they all point in the same positive direction. The strongest within-subject correlation of *r* = 0.34 was found by fusing all three sensor modalities. Notice that within-subject correlations are approximately the same using only eye-tracking and speech. Adjusted correlations, $\hat{r}$, were also computed, to adjust for the fact that the variance of *dU* can be smaller than that of *U*. The adjusted correlations have similar magnitudes as the raw correlations, indicating that range restriction has little effect.

**Table 7:** Latent space correlations between *dU* and *dV* given individual feature modalities and fused modalities as input (*n* = 40).

**Modality**	***dU**, **dV***		
	**r**	$\hat{r}$	**p**
1. Eye	0.28	0.26	0.078
2. Speech	0.11	0.11	0.506
3. fMRI	0.23	0.24	0.152
1, 2	0.33	0.32	0.039
1, 3	0.20	0.22	0.217
2, 3	0.21	0.24	0.192
1, 2, 3	0.34	0.40	0.034

*dU and dV are computed from successive within-subject sessions. Also shown are correlations, $\hat{r}$, that are adjusted for range restriction in dU relative to U*.

## 6. Discussion

The goal of this work was to investigate the usefulness of biomarkers for estimating cognitive performance outcomes. The subject population was high school athletes participating in sports (football and soccer) that are associated with elevated risk of concussion and of subconcussive head impacts. Cognitive performance was measured using the ImPACT diagnostic test. The biomarkers were obtained in three sensing modalities: eye movement, speech audio, and brain fMRI. Each of the sensing modalities has shown current, or emerging, capability for providing passive, ambulatory data capture during normal activity. Using a predictive model trained and tested with leave-one-subject-out cross-validation, we found strong predictions from the fused sensor modalities of a latent cognitive performance factor (*r* = 0.71), with a weaker ability to predict within-subject changes in this factor over time (*r* = 0.34).

### 6.1. Clinical Relevance

Particular patterns of correlations were found between the ImPACT latent factor and coordination complexity features from each sensor modality. These patterns show that better cognitive performance is associated with more precise (and hence lower dimensional) smooth pursuit eye movements. This is seen in Figure 3right where the negative correlations in eigenvalue ranks 3–26 indicate that lower eye movement variability (i.e., smaller values in these eigenvalue ranks) is associated with higher cognitive performance. Increased variability of smooth pursuit eye movements have been associated with mTBI (52, 53). Therefore, it is plausible that a loss of cognitive performance due to mTBI would be manifested in changes in the smooth pursuit coordination complexity features.

With dFormant speech features, the correlation patterns are more complex. In Figure 4right, there are positive correlations with cognitive performance in eigenvalues in ranks 9–16, followed by negative correlations in higher rank eigenvalues. This indicates that higher cognitive performance is associated with more complex articulatory movements at a particular range of temporal frequencies, as coded by the eigenvectors of ranks 9–16, with less complexity at a higher range of temporal frequencies.

With subcortical fMRI features, there are positive correlations with cognitive performance in eigenvalues of rank 15–95. This correlation pattern indicates that subjects with higher cognitive scores produce brain activation dynamics with more complexity, in the sense that they contain a greater fraction of their temporal variability in the measurement-space directions encoded by eigenvectors in these ranks.

Thus, a reasonable interpretation of these results, in aggregate, is that better cognitive performance is associated both with the generation of more precise motor movements and with greater complexity in resting state brain dynamics. With regard to motor movements, higher precision can cause simpler motor trajectories in some contexts but more complex trajectories in others. For simple directed movements (eye tracking), higher precision causes more accurate execution of simple trajectories. For complex movements (speech), higher precision causes a more accurate execution of complex trajectories. Moreover, the results in section 5.5 provide some support for the hypothesis that within-subject cognitive performance changes are reflected in biomarker changes in a manner similar to how between-subject performance differences are reflected in biomarker differences.

### 6.2. Limitation and Future Work

One limitation of this work is the absence of any documented mTBIs in the sample at the time the signals were recorded and ImPACT testing was conducted. Thus, the dataset should be viewed as a convenience sample in which longitudinal cognitive performance measurements were obtained simultaneously with biomarker measurements, and the primary findings reflect variability and changes in cognitive performance broadly. Indeed, there are a number of factors, both individual and environmental, that could have influenced subjects' performance on the ImPACT test, including the effects of sports-related head impacts. However, we are not currently able to disambiguate these. At this early stage in the development of noninvasive measures of cognitive state changes subsequent to head impacts, we are only able to verify that, with the current dataset, we can specify which ImPACT scores correlate with our sensor-based features, how they correlated, and how much of that correlation is within-subject vs. across-subject. The next step for this work is to determine whether these correlation patterns can also serve as indicators of concussion status and/or head impact biomechanics.

A possible confound is the use of two different subject populations: a cohort of male 22 football players, and one of 6 female soccer players. A Mann-Whitney ranksum test was conducted to determine if there were systematic differences in the ImPACT Composite scores for the two subject groups. For each of the six composites, the difference between the two groups was insignificant (*p*>0.05). This finding reduces the risk that relationships which were found between sensor measurements and ImPACT scores were confounded by group-level differences.

Another limitation is that the size of the data set size limits the ability to discover statistically significant relationships between sensor measurements and cognitive performance outcomes. The machine learning method used in this paper, CCA, provides the ability to assess how well the neuromotor coordination features predict additional (linear) dimensions of variation in the pattern of ImPACT scores. It was found that adding additional latent dimensions did not improve accuracy in predicting ImPACT composite scores on out of sample subjects. However, with a larger dataset and using nonlinear machine leaning methods, the possibility remains that additional modes of variation in impact performance may be explainable from the coordination complexity features.

New technology provides the opportunity to capture large volumes of data in free living conditions, and to discover relationships between sensor measurements and important behavioral outcomes such as cognitive performance. These relationships can provide a foundation for promptly detecting subtle decrements in performance due to injury.

With smooth pursuit eye tracking, it is plausible that the current laboratory findings will generalize to eye tracking movements collected in field conditions. It has been found that the mean and variation of visually driven smooth pursuit can be accounted for by properties of the sensory representation of visual motion in extrastriate visual area MT, with movements modulated by sensory-motor and motor circuits in the cerebellum and the smooth eye movement region of the frontal eye fields (17). Better neurological functioning should correlate with better ability to fixate and track objects in the world. This, in turn, will tend to be manifested in smoother, more parsimonious, eye movement tracking trajectories.

Measuring smooth pursuit eye movements requires taking into account simultaneous head movements. Therefore, this technology will benefit from the incorporation of head-mounted inertial measurment devices (IMUs) that simultaneously estimate head position and head movements.

Free speech has shown great promise in providing information about cognitive state related to mTBI, comparable in quality to read speech (21). Therefore, it is recommended that read speech protocols should be augmented with naturally occurring speech with speaker diarization. In addition, formant speech features should be augmented with alternative feature modalities, including pitch, intensity envelope, and mel-frequency cepstral coefficients, which have shown great promise in detecting the presence of altered cognitive state due to mTBI (21).

Measuring brain activity in free living conditions is a less mature technology. Our findings are based on fMRI measurements, showing differences in resting state connectivity that are measured while subjects are not moving and not involved in cognitive tasks. It is notable that our strongest fMRI findings involve coordination complexity features, which, because they are based on temporal correlations, do not rely on calibrated signals that accurately measure absolute brain blood flow levels or neural activity levels. Rather, they only require that the time series indicate temporal fluctuations in these levels.

An important factor in fMRI analysis is the choice of atlas registration for computing ROI time series from raw fMRI signals (see Tables 2, 3). With our particular choice of atlas, stronger correlations were obtained from subcortical ROIs than cortical ROIs. One possible cause of this discrepancy is the fact that the subcortical atlas contains separate ROIs for each hemisphere, whereas the cortical atlas does not. Investigating choice of atlas, as well as possible sub-selection of discriminative ROIs given choice of atlas, are areas for further research.

## 7. Conclusion

Repeated insults to the head, including those categorized as sub-concussive, can potentially cause lasting neurological changes. Dynamics of smooth pursuit eye movements, speech production, and resting state brain activity may provide sensitive indicators of neurological changes. A first step in validating this relationship is to show that these dynamics correlate with objective measures of cognitive performance. This paper demonstrates that features characterizing the dynamics of eye movements, speech, and brain activity show strong correlations with cognitive performance measures from the ImPACT test, including moderate correlations with within-subject changes in those cognitive performance measures. These results provide a step toward the development of objective indicators of neurological health that could be used for early warning of neurological damage due to repeated sub-concussive injuries.

## Data Availability Statement

The data analyzed in this study is subject to the following licenses/restrictions: The data may be available on request, subject to IRB approval. Requests to access these datasets should be directed to James Williamson, jrw@ll.mit.edu.

## Ethics Statement

The studies involving human participants were reviewed and approved by Committee on the Use of Humans as Experimental Subjects (COUHES), which acts at the Institutional Review Board for the Massachusetts Institute of Technology. Written informed consent to participate in this study was provided by the participants' legal guardian/next of kin.

## Author Contributions

TV, JL, TS, and TT performed data collection. JW and DS analyzed the data. JW, DS, and SY wrote the manuscript. HR, KH, and TT provided feedback on the analysis and manuscript. TQ oversaw all aspects of the program. All authors contributed to the article and approved the submitted version.

## Conflict of Interest

The authors declare that the research was conducted in the absence of any commercial or financial relationships that could be construed as a potential conflict of interest.
